# Supplementary material for: Multi‐dataset identification of innovative feature genes and molecular mechanisms in keratoconus
Source: J Cell Mol Med. 2024 Sep 19;28(18):e70079. doi: 10.1111/jcmm.70079 (PMC11412914; doi:10.1111/jcmm.70079)
Supplement: Supplementary file 2 — Material S2. [file JCMM-28-e70079-s002.zip › 7.GSEA analysis/3.DBNDD1/KEGG_NON_SMALL_CELL_LUNG_CANCER.html]

Details for gene set KEGG\_NON\_SMALL\_CELL\_LUNG\_CANCER[GSEA]

|  || Dataset | DBNDD1.group.cls#h\_versus\_l |
| Phenotype | group.cls#h\_versus\_l |
| Upregulated in class | h |
| GeneSet | KEGG\_NON\_SMALL\_CELL\_LUNG\_CANCER |
| Enrichment Score (ES) | 0.15695508 |
| Normalized Enrichment Score (NES) | 0.7214758 |
| Nominal p-value | 0.84836066 |
| FDR q-value | 1.0 |
| FWER p-Value | 1.0 |
Table: GSEA Results Summary

  

Fig 1: Enrichment plot: KEGG\_NON\_SMALL\_CELL\_LUNG\_CANCER      
 Profile of the Running ES Score & Positions of GeneSet Members on the Rank Ordered List

  

| SYMBOL | TITLE | RANK IN GENE LIST | RANK METRIC SCORE | RUNNING ES | CORE ENRICHMENT || 1 | PIK3R5 | na | 35 | 0.708 | 0.1570 | Yes |
| 2 | RARB | na | 2894 | 0.256 | 0.1317 | No |
| 3 | PRKCG | na | 6020 | 0.150 | 0.0750 | No |
| 4 | E2F2 | na | 6645 | 0.133 | 0.0867 | No |
| 5 | PIK3R1 | na | 6912 | 0.126 | 0.1072 | No |
| 6 | SOS1 | na | 8586 | 0.093 | 0.0796 | No |
| 7 | AKT2 | na | 9388 | 0.080 | 0.0744 | No |
| 8 | CCND1 | na | 9676 | 0.076 | 0.0830 | No |
| 9 | MAPK3 | na | 9865 | 0.073 | 0.0939 | No |
| 10 | RB1 | na | 10147 | 0.069 | 0.1013 | No |
| 11 | MAP2K2 | na | 10931 | 0.059 | 0.0919 | No |
| 12 | FHIT | na | 11606 | 0.051 | 0.0839 | No |
| 13 | PDPK1 | na | 11670 | 0.050 | 0.0933 | No |
| 14 | PIK3R2 | na | 11699 | 0.050 | 0.1036 | No |
| 15 | PLCG1 | na | 11955 | 0.048 | 0.1069 | No |
| 16 | RXRA | na | 12017 | 0.047 | 0.1156 | No |
| 17 | ERBB2 | na | 13132 | 0.037 | 0.0918 | No |
| 18 | SOS2 | na | 13580 | 0.033 | 0.0863 | No |
| 19 | TP53 | na | 13962 | 0.030 | 0.0821 | No |
| 20 | KRAS | na | 14127 | 0.029 | 0.0839 | No |
| 21 | PIK3R3 | na | 14144 | 0.029 | 0.0898 | No |
| 22 | BRAF | na | 15471 | 0.019 | 0.0559 | No |
| 23 | RASSF5 | na | 16344 | 0.014 | 0.0338 | No |
| 24 | PIK3CA | na | 17073 | 0.009 | 0.0148 | No |
| 25 | PIK3CB | na | 18338 | 0.000 | -0.0216 | No |
| 26 | STK4 | na | 18367 | 0.000 | -0.0223 | No |
| 27 | HRAS | na | 18984 | -0.004 | -0.0391 | No |
| 28 | RAF1 | na | 19005 | -0.005 | -0.0387 | No |
| 29 | AKT1 | na | 19066 | -0.005 | -0.0393 | No |
| 30 | EGF | na | 19333 | -0.007 | -0.0454 | No |
| 31 | PLCG2 | na | 19340 | -0.007 | -0.0439 | No |
| 32 | CASP9 | na | 19881 | -0.012 | -0.0568 | No |
| 33 | BAD | na | 20453 | -0.017 | -0.0695 | No |
| 34 | CDK4 | na | 21131 | -0.023 | -0.0838 | No |
| 35 | CDKN2A | na | 21295 | -0.025 | -0.0830 | No |
| 36 | PRKCA | na | 21412 | -0.026 | -0.0806 | No |
| 37 | RXRB | na | 21455 | -0.026 | -0.0759 | No |
| 38 | EGFR | na | 22041 | -0.033 | -0.0855 | No |
| 39 | GRB2 | na | 22294 | -0.035 | -0.0850 | No |
| 40 | MAPK1 | na | 22705 | -0.040 | -0.0879 | No |
| 41 | TGFA | na | 22844 | -0.041 | -0.0827 | No |
| 42 | RASSF1 | na | 23202 | -0.046 | -0.0828 | No |
| 43 | FOXO3 | na | 23455 | -0.049 | -0.0792 | No |
| 44 | E2F3 | na | 24031 | -0.057 | -0.0831 | No |
| 45 | ARAF | na | 24817 | -0.068 | -0.0906 | No |
| 46 | PIK3CD | na | 24971 | -0.070 | -0.0793 | No |
| 47 | NRAS | na | 27014 | -0.108 | -0.1141 | No |
| 48 | CDK6 | na | 27414 | -0.117 | -0.0995 | No |
| 49 | PIK3CG | na | 27455 | -0.118 | -0.0743 | No |
| 50 | E2F1 | na | 27800 | -0.126 | -0.0561 | No |
| 51 | RXRG | na | 28564 | -0.145 | -0.0457 | No |
| 52 | MAP2K1 | na | 29246 | -0.164 | -0.0287 | No |
| 53 | AKT3 | na | 33656 | -0.385 | -0.0700 | No |
| 54 | PRKCB | na | 34153 | -0.452 | 0.0166 | No |
Table: GSEA details [plain text format]

  

Fig 2: KEGG\_NON\_SMALL\_CELL\_LUNG\_CANCER      
 Blue-Pink O' Gram in the Space of the Analyzed GeneSet

  

Fig 3: KEGG\_NON\_SMALL\_CELL\_LUNG\_CANCER: Random ES distribution      
 Gene set null distribution of ES for **KEGG\_NON\_SMALL\_CELL\_LUNG\_CANCER**
